# Phage Therapy: Beyond Antibacterial Action

**DOI:** 10.3389/fmed.2018.00146

**Published:** 2018-05-23

**Authors:** Andrzej Górski, Ewa Jończyk-Matysiak, Ryszard Międzybrodzki, Beata Weber-Dąbrowska, Marzanna Łusiak-Szelachowska, Natalia Bagińska, Jan Borysowski, Małgorzata B. Łobocka, Alicja Węgrzyn, Grzegorz Węgrzyn

**Affiliations:** ^1^Bacteriophage Laboratory, Hirszfeld Institute of Immunology and Experimental Therapy, Polish Academy of Sciences, Wrocław, Poland; ^2^Phage Therapy Unit, Hirszfeld Institute of Immunology and Experimental Therapy, Polish Academy of Sciences, Wrocław, Poland; ^3^Department of Clinical Immunology, Transplantation Institute, Medical University of Warsaw, Warsaw, Poland; ^4^Institute of Biochemistry and Biophysics, Polish Academy of Sciences, Warsaw, Poland; ^5^Autonomous Department of Microbial Biology, Faculty of Agriculture and Biology, Warsaw University of Life Sciences, Warsaw, Poland; ^6^Laboratory of Molecular Biology, Institute of Biochemistry and Biophysics, Polish Academy of Sciences, Gdańsk, Poland; ^7^Department of Molecular Biology, University of Gdańsk, Gdańsk, Poland

**Keywords:** phage, phage therapy, immunomodulation, inflammation, prophage induction

## Abstract

Until recently, phages were considered as mere “bacteria eaters” with potential for use in combating antimicrobial resistance. The real value of phage therapy assessed according to the standards of evidence-based medicine awaits confirmation by clinical trials. However, the progress in research on phage biology has shed more light on the significance of phages. Accumulating data indicate that phages may also interact with eukaryotic cells. How such interactions could be translated into advances in medicine (especially novel means of therapy) is discussed herein.

## Introduction

Anti-microbial resistance (AMR) has been recognized as a fundamental threat to human health and one of the greatest challenges to our civilization; it is estimated that by 2050 ten million people may be dying annually and the economic burden may hit $100 trillion (1). This crisis has greatly revived interest in bacteriophages (phages), viruses of bacteria, as a potential weapon against AMR which could be used to treat patients with infections caused by antibiotic-resistant bacteria. More than 10 reviews addressing different aspects of phage therapy (PT) have been published in the past year; recently, *The Lancet* (2), *JAMA* (3) and *Science* discussed the progress in PT (4). This well illustrates the growing interest in the potential of PT in parallel to an increase in the threat of AMR. In the past year significant progress in the application of PT in broader clinical practice was noted when successful intravenous application of phages in individual patients in Belgium and USA was described (5–7).

While the real therapeutic value of PT still awaits confirmation by clinical trials, the progress in research on immunobiology of phages has supplied new and exciting data which confirm our notion that PT has “potential for evolving from merely a treatment for complications to targeting diseases” (8). Thus, in addition to eliminating bacteria PT might also be considered to control inflammation including that occurring in the course of some non-communicable diseases. These anti-inflammatory and immunomodulating effects of phages have recently been discussed in detail (9).

Furthermore, there has been a significant broadening in our knowledge of phage presence within the human body and possible significance of this phenomenon in health and disease. Phages are present in high concentrations in the intestinal tract and can often be detected in ascitic fluid, urine and saliva (10, 11). Progress in metagenomic analysis has also provided data to indicate that phages are not only present in oropharyngeal and urinary samples but may also circulate in blood (12–14).

The largest number and diversity of phages can be found in the gut. We have postulated that intestinal phages may translocate from the gut into lymph, blood and tissues, mediating immunomodulatory functions (15). These assumptions have recently been confirmed and extended by Barr et al., who also put forward the concept of the “intrabody phageome” involving phages capable of bypassing epithelial cell layers, disseminating throughout the body and influencing the immune system (16, 17). The authors suggest that as many as 31 billion phages undergo transcytosis from the gut each day, thus contributing to the “bacteriophage journey through the human body” (16). Moreover, these data have recently been extended by other authors who showed that certain phages could bind to polysialic acid (a eukaryotic cell surface glycan responsible for cell interactions) and penetrate human neuroblastoma cells, where they are detectable within the perinuclear region of the cells (no penetration of phage DNA into the nucleus was detectable). Thus, “phages cross the border to eukaryotes” (18). The ability/inability of phage DNA to penetrate into the nucleus may depend on the phage. Terminal proteins of phages that use protein-primed replication to amplify their DNA commonly contain nuclear localization signals (19, 20). When they are expressed in mammalian cells they can covalently attach to the 5′ ends of linear DNA molecules and enhance the delivery of these molecules to the nucleus. A functional nuclear localization signal was also identified in the P1 phage-encoded recombinase Cre, which serves as a standard tool for *in vivo* manipulations with prokaryotic and eukaryotic chromosomes (21).

## Bacteriophage abundance in human intestine

Two kinds of phages occupy the human gut as a specific environment. One group consists of virulent (lytic) phages which develop solely according to the lytic pathway, in which infected bacteria are eventually lysed and phage progeny are released from the host cell. Filamentous phages that do not cause true lysis of the host cell but produce progeny which escape the bacterium in a budding-like process for a relatively long time could be considered as a specific kind of virulent phages in this discussion. The second group contains temperate phages that are able to develop either lytically or according to the lysogenic mode. The latter process consists of integration of the phage genome into the host chromosome or establishment of the phage genome in the form of a plasmid. Irrespective of the kind of lysogenization, the prophage is able to replicate the phage genome in the host cell, either together with the chromosome or as a plasmid. During lysogeny only genes encoding prophage maintenance functions and so-called lysogenic conversion genes are expressed. The latter are responsible for the protection of the lysogen from infection by similar or certain other phages or encode other functions adaptive for bacteria to counteract the disadvantage of the extra DNA load, which may cause outcompetition of lysogens by faster replicating prophage free cells. Under specific conditions, a prophage can be induced, which results in excision of its genome from the host chromosome (if the prophage is not propagated as a plasmid) and entry into the lytic developmental pathway [for a general overview, see (22)]. It should be kept in mind that the gut bacteria harbor a large number of prophages whose biological role is unclear, while prophage production may be approximately 200 × higher *in vivo* in the intestines than *in vitro* (23). In fact, the majority of gut commensal bacteria are lysogens, of which most are active, and prophages are spontaneously induced as active phages (24).

## Mechanisms of prophage induction *in vitro* and *in vivo*

The process of prophage induction can be either spontaneous, resulting from variations in efficiency of expression of genes involved in prophage maintenance, or caused by various factors. The observed frequency of spontaneous prophage induction is relatively low. In studies with an *Escherichia coli* polylysogen they varied from <1 per 10^5^ cells to 5 per 10^4^ cells (25). In general, lysogeny is the predominant form of temperate phage propagation (26–31). Various chemical or physical factors might induce a prophage in most, or if not all, lysogenic cells in a bacterial population, causing effective phage lytic development and production of a large number of phage progeny [for a review see (32)]. The general problem is, however, that despite our extensive knowledge about molecular mechanisms of the genetic switch between lysogenic and lytic modes of development of a few model bacteriophages (32–35), understanding of the same process occurring in the human intestine is relatively poor. In fact, the best investigated factors responsible for prophage induction in different lysogenic bacteria are expected to be either unlikely (UV irradiation) or rarely occurring (high concentrations of antibiotics which affect DNA structure directly or indirectly) in the human gut (32–35). Therefore, although studies on such factors, which indicated that efficient prophage induction usually requires activation of the RecA protein and resultant S.O.S. response, were crucial to understand the mechanism of this phenomenon, they could not provide information about its efficiency in lysogenic bacteria present in the intestine. Only recent reports indicated what agents may cause prophage induction there.

Studies that focused on prophage induction under conditions resembling those occurring in the human gut usually concerned bacteria bearing prophages which contain genes coding for toxins or other virulent agents. When looking for chemicals which can induce Shiga toxin-converting prophages, it was found that hydrogen peroxide (HP) might be an efficient prophage induction agent (36, 37). However, the highest observed efficiency of this process did not exceed a few percent of cells in which H_2_O_2_-mediated prophage induction occurred (36, 38). This is in contrast to over 10-times higher efficiency of prophage induction caused by low concentrations of mitomycin C, an antibiotic that interacts directly with DNA (39). However, the discovery that H_2_O_2_ is a prophage inductor was important, as this compound can occur in the human intestine, particularly when neutrophils recognize a bacterial infection, migrate to the intestine and excrete this chemical as an antibacterial agent (40, 41). In fact, gut inflammation may increase prophage induction 10^5^ fold (42). HP occurs in seawater and may be an inducing agent in nature; its concentrations in water correspond to peak natural levels (0.1–1.35 μM) (43). Other experiments performed *in vitro* indicated that some other factors or conditions that might potentially be present (either often or rarely) in the human gut can induce prophages to some extent. They include low pH (<4) (38, 44), increased concentrations of sodium chloride (2–3%) (45, 46) or bile salts (44), monovalent cations (46), chelators of Mg^2+^ ions such as EDTA or citrate (47, 48), disodium phosphate (48), copper (49), and cyanide (49). There are also reports showing that some physical conditions might also cause prophage induction, for instance irradiation with ^60^Co (50), high hydrostatic pressure (51) or a 50 Hz rotating magnetic field (52). Moreover, temperature can modulate phage development significantly (40). Processes of stimulated prophage induction have been found in various bacterial species, including *Escherichia coli, Salmonella enterica, Streptococcus pneumoniae, Helicobacter pylori, Lactococcus lactis, Clostridium difficile* and others (32, 39, 53). Importantly, recent preliminary studies demonstrated that some commonly used drinks, such as ice tea, may also induce prophages at concentrations likely occurring in the human gastrointestinal tract. This suggests that nutrients can influence bacteriophage biology significantly (54). Furthermore, prophage induction may occur as a result of interactions with some cells (e.g., fibroblasts, pharyngeal epithelial cells) (55, 56). Finally, immunosuppressive drugs (e.g., cyclosporine A, mycophenolic acid) induce oxidative stress and therefore could also contribute to prophage induction in treated patients (57–59).

An additional factor that cannot be overlooked when considering prophage induction is the increasing contamination of the environment by xenobiotics, including carcinogens. Several chlorinated phenols and chlorinated pesticides have been shown to induce the lambda prophage (60, 61). Chlorinated phenols and their conjugates are ingested with food and drinking water. They can also penetrate skin. They were detected in high concentrations in the bile of trout (62). Exposure of goldfish (*Carassius auratus*) to pentachlorophenol (PCP) caused significant changes in the *Firmicutes*/*Bacteroides* ratio in the fish gut (63). The range of observed changes was correlated with PCP exposure dosage and duration. In other studies, metabolic activation of PCP was shown to lead to prophage induction (64). Carcinogenicity/genotoxicity testing of various substances, including hazardous industrial waste, using *c*I*ts*857 or wild-type prophage induction as an indicator, was found to be more sensitive than the traditional Ames *Salmonella*/microsome test, indicating that certain xenobiotics or carcinogenic agents in the gastrointestinal tract as well as in other body niches may cause prophage induction at lower concentrations than those leading to major changes in microbiome composition (65, 66). In the case of 11 out of 14 wastes tested, the induction occurred at concentrations as low as 0.4 pg/ml.

Bacterial and viral communities in the mammalian gut were shown in several studies to be diet-specific (67). Moreover, more or less pronounced changes in the gut microbiome appear to be a hallmark of gastrointestinal disorders and certain autoimmune disorders as well [see e.g., (68)]. For instance, comparative studies of metagenomic samples from patients with type 1 diabetes (T1D) and healthy individuals revealed significantly higher representation of phages and prophages in patients with this disorder, as compared to the control group (68). Qualitative or quantitative changes within the gut microbiome are inevitably associated with changes in the gut virome. First, different bacteria carry different prophages, and thus the repertoire of gut phages that are released upon induction of prophages depends on the repertoire of gut bacteria. Second, density changes within component populations of mixed microbial communities may turn on or off quorum sensing mechanisms. Signaling molecules of quorum sensing such as acyl homoserine lactones (AHL) could cause the induction of lambda prophage, when produced by *Pseudomonas aeruginosa* PAO1 strain in a coculture with *E. coli* lysogen of lambda (69). AHL could induce phage production also in soil and groundwater bacteria, implying its similar action in other environments. Spontaneously arranged forms of 4,5-dihydroxy-2,3 pentanedione—an unstable bacterial metabolic product, called collectively autoinducer-2 (AI-2)—are thought to be universal quorum sensing signals for many bacterial species (70–74). Representatives of a given bacterial species are able to recognize a particular molecule from among the AI-2 pool. AI-2 can act as an inducer of expression of several genes and mutual biofilm formation by bacteria in a concentration-dependent manner. It was hypothesized to act as an interspecies signal that can interact with structurally and functionally different receptors of various bacterial species (75). In *Enterococcus faecalis*—one of the gut commensals—AI-2 is used as a signaling molecule that can cause prophage induction regulating biofilm formation (76). In addition to AI-2, prophage induction in enterococci occurs upon treatment with certain antibiotics, such as ciprofloxacin (77, 78). Both situations may occur in the gastrointestinal tract of hospitalized neutropenic patients, who commonly receive quinolone antibiotics, leading to selective enrichment of their intestinal microflora with enterococci (76). Released phages are likely to infect susceptible bacteria in the intestine, facilitating horizontal gene transfer and qualitative changes in the intestine virome.

The question remains how many bacteriophages can be present in the human intestine, considering propagation of virulent phages and induction of prophages, followed by their lytic development. Knowing that the average number of bacterial cells in the human gut is 3.8 × 10^13^, as recently estimated (79), even assuming that the efficiency of prophage induction under natural conditions by a single agent is low, and occurs in only a small fraction (from 0.001 to 3%) of lysogenic cells (39, 54), the number of bacteriophages present in the gut must still be significant. When measuring the number of virions of just one kind of bacteriophage in human fecal samples, 2.6 × 10^4^ phages per g of stool were found (80). Thus, there were perhaps at least 10^7^ virions of this particular phage in the whole intestine, and it is worth mentioning that a temperate phage was considered without evident prophage induction conditions. When trying to estimate the number of phages *in vivo*, it is crucial to mention that using a mouse model, it was calculated that the frequency of prophage induction in the mammalian intestine is about 1,000-fold higher than that measured in *in vitro* experiments (81). Therefore, the recent estimation that the human gastrointestinal tract may contain about 10^15^ virions of bacteriophages (82) indeed appears realistic. In support of that, in metagenomic studies of the microbiome and virome of mice, only about 10% of reads could be assembled into a bacterial genome, while most of the remaining reads were assembled into viral genomes, with the dominant fraction being assembled into phage genomes (67). If so, in the human body, bacteriophages are abundant enough to cause significant biological effects on reactions which they may either affect significantly or even modulate moderately.

## “Natural phage therapy”

Data indicating that phages may pass the intestinal epithelia, circulate in blood and penetrate other cells and tissues raise many important questions about the significance of this phenomenon. Critical analysis of data available from *in vitro* and *in vivo* studies strongly suggests that phages do not exert significant harmful effects on eukaryotic cells. Furthermore, results of experimental phage therapy including hundreds of patients in Georgia, Poland, Russia (as well as the most recent data from intravenous treatment of single patients in Belgium and USA) do not raise significant concern about phage toxicity to the mammalian (and in particular human organism, including children). However, those findings do not exclude the possibility that phage penetration into mammalian cells may modify their functions. Laboratory data derived from patients on phage therapy do not indicate that phages exert harmful effects on the function of internal organs, bone marrow or skin; on the other hand, they can induce reactions from the immune system (e.g., antibody production); moreover—as stated previously—phages may modulate immune functions. This is not entirely unexpected—for example, it is known that phages are taken up and processed by phagocytes, so they could modify the functions of those cells. Data available so far suggest that the prevailing effect of phages is anti-inflammatory and downregulating immune hyperactivity, which offers hope for phage applications in medicine beyond their well-known antibacterial action (perhaps also in autoimmune disease, allograft rejection, etc.). In fact, two recent commentaries in *Science* suggest that phages present in the human body “keep us healthy” and “protect our health” (4). Those data and suggestions fit well with our hypothesis that envisions such a role for endogenous phages, protecting our bodies from internal and external enemies, while such safeguarding could be possible not only *in situ* but also in other tissues as a result of phage translocation from the intestinal tract (“natural phage therapy”) (15, 83, 84).

The fact that phages may combine their anti-bacterial action with anti-inflammatory and immunoregulatory effects suggests that their potential therapeutic applications may acquire new dimensions well beyond mere elimination of bacteria. If so, this new concept of phage therapy could offer new perspectives for treating so far poorly controlled disorders in which there exists no targeted therapy or the currently available therapies have significant side effects. Sepsis constitutes an excellent example of the potential of phage therapy in this clinical setting where phages can eliminate offending bacteria and downregulate inflammation (sepsis is believed to be a complex pathology where an uncontrolled inflammatory response to bacterial infection constitutes a hallmark of that pathology). In particular, the ability of phages to bind endotoxins, inhibit excessive reactive oxygen species production, and diminish inflammatory infiltration of skin and internal organs, combined with their confirmed ability to lower clinical and laboratory indices of inflammation, may be especially relevant in treating the sepsis syndrome. PT may be beneficial if administered early in sepsis, as immunosuppression is typical for its later stages (85, 86). However, it appears that the immunomodulatory activity of phages may be applied in the clinic according to the principles of personalized medicine as the final effect depends on an initial immune status of a patient (87). On the other hand, PT appears to be safe when applied in immunodeficiency syndromes (88) and, very interestingly, upregulation of phagocytosis has been associated with good prognosis of PT (87). This is an important argument for more studies on PT effectiveness in sepsis as stimulation of phagocytosis is recommended as one of the most promising approaches which may significantly improve prognosis of treatment of this serious complication (89).

According to our vision, phages could also interact with intestinal epithelial cells (IEC). For example, IEC constitutively express an anti-inflammatory cytokine Il-1 receptor antagonist (Il1RN). Phages can upregulate the synthesis of Il1RN; therefore they could counterbalance increased production of Il-1 in intestinal inflammatory disorders. Also, phage-dependent reduction of reactive oxygen species (ROS) production could be beneficial in inflammatory bowel diseases (IBD), where oxidative stress is believe to contribute to their pathology. What is more, phage may induce Il-10 production, known to play an important role in intestinal homeostasis. This effect could be similar to the action of probiotics that also upregulate Il-10 secretion by phagocytes in the gut. These associations between the immunopathology of IBD and the immunomodulatory action of phages prompted us to formulate a hypothesis on potential application of phages in immunotherapy of IBD (90).

We have hypothesized that phages may control immune homeostasis by their interactions with liver macrophages (Kupffer cells, KC). KC are known to induce immunological tolerance via Il-10, which those cells produce; deletion of KC suppresses liver damage in experimental hepatitis (91). In addition, KC are involved in the phenomenon of liver allograft tolerance (92). Moreover, Il-10-mediated suppression of ROS and inflammatory cytokines is induced when KC phagocytosis is increased (93). Interestingly, the liver is a primary site of phage uptake following their parenteral administration (94). Those findings have prompted us to suggest that PT targeting liver KC may be considered as a potential immunotherapy in autoimmune liver disease (95).

Perhaps the most provocative hypothesis that we have recently proposed focuses on the possible application of PT in allergy. Here again, phage-induced Il-10 upregulation plays a paramount role, as this cytokine has been demonstrated to reduce the number of eosinophils and mast cells and to stabilize and prevent antigen-mediated activation of these cells. Moreover, specific immunotherapy increases Il-10 production and causes IgE to switch toward normal immunity (96). Furthermore, Il1RA (whose level may be upregulated by phages) may reduce allergen-dependent airway inflammation and experimentally induced allergic eye disease (97). Recently, successful use of PT has been reported in a boy with atopic diathesis (98). Importantly, PT does not cause an increase in the number of eosinophils in blood of patients (99). Interestingly, in 1956 Mills suggested that staphylococcal phage preparations may have anti-allergic effects in patients with sinusitis (100). Therefore, we believe that there exist sound reasons to explore the option of potential use of PT in allergy treatment (101).

In the past years our current understanding of the role of phages in nature and their potential in medicine has shifted from mere “viruses of bacteria” that influence the number and functions of bacteria and could be used to combat bacterial infections to the notion of an important component of our organisms abundantly present in the intestines, from where phages can migrate to blood and tissues. Thus, it is becoming evident that phages can interact not only with bacteria but also with eukaryotic cells. Research on the significance of such phage-eukaryotic cell interactions is at a very early stage. However, data obtained so far strongly suggest that phages may induce immunomodulatory effects which can be used in the clinic in PT. Moreover, these novel findings highlight the potential protective role of “endogenous” phages present in the human body (“natural PT”). In this sense, a better understanding of mechanisms responsible for prophage induction *in vivo* and the significance of such phages may yield new and exciting data, including the possible role of HP in regulating this process in health and disease.

One way or another, the doors to further research on phage–eukaryotic cell interactions are open wide. We do not suggest that PT will be a panacea. However, the potential for broader application of PT is evident and it is certainly worthy of further studies.

## Author contributions

AG drafted the main part of the manuscript. EJ-M, RM, BW-D, MŁ-S, NB, JB, MŁ, AW, and GW contributed parts of the manuscript. All authors approved the manuscript.

### Conflict of interest statement

AG, RM, BW-D, JB, and MŁ are co-inventors of patents owned by the Hirszfeld Institute of Immunology and Experimental Therapy and covering phage preparations. The other authors declare that the research was conducted in the absence of any commercial or financial relationships that could be construed as a potential conflict of interest. The reviewer ER and handling Editor declared their shared affiliation.
